# Hypomethylation in *MTNR1B*: a novel epigenetic marker for atherosclerosis profiling using stenosis radiophenotype and blood inflammatory cells

**DOI:** 10.1186/s13148-023-01423-x

**Published:** 2023-01-19

**Authors:** Jee Yeon Kim, Jaroslav Jelinek, Young Ho Lee, Dae Hyun Kim, Keunsoo Kang, Su Hyun Ryu, Hye Rin Moon, Kwangjo Cho, Seo Hee Rha, Jae Kwan Cha, Jean-Pierre J. Issa, Jei Kim

**Affiliations:** 1 Department of Neurology and Neuroepigenetics Laboratory, College of Medicine and Hospital, Chungnam National University, 282 Moonhwaro, Joongku, Daejeon, 35015 South Korea; 2 Coriell Institute for Medical Research, Camden, NJ USA; 3 Department of Anatomy, College of Medicine, Chungnam National University, Daejeon, South Korea; 4 Department of Neurology, Dong-A University Hospital, Busan, South Korea; 5 Department of Microbiology, College of Science and Technology, Dankook University, Cheonan, South Korea; 6 Department of Thoracic and Cardiovascular Surgery, Dong-A University Hospital, Busan, South Korea; 7 Department of Pathology, Dong-A University Hospital, Busan, South Korea; 8 Daejeon-Chungnam Regional Cerebrovascular Center, Chungnam National University Hospital, Daejeon, South Korea

**Keywords:** Atherosclerosis, Promoter methylation profiling, Stenosis radiophenotype, Blood inflammatory cells, Radioepigenomics

## Abstract

**Background:**

Changes in gene-specific promoter methylation may result from aging and environmental influences. Atherosclerosis is associated with aging and environmental effects. Thus, promoter methylation profiling may be used as an epigenetic tool to evaluate the impact of aging and the environment on atherosclerosis development. However, gene-specific methylation changes are currently inadequate epigenetic markers for predicting atherosclerosis and cardiovascular disease pathogenesis.

**Results:**

We profiled and validated changes in gene-specific promoter methylation associated with atherosclerosis using stenosis radiophenotypes of cranial vessels and blood inflammatory cells rather than direct sampling of atherosclerotic plaques. First, we profiled gene-specific promoter methylation changes using digital restriction enzyme analysis of methylation (DREAM) sequencing in peripheral blood mononuclear cells from eight samples each of cranial vessels with and without severe-stenosis radiophenotypes. Using DREAM sequencing profiling, 11 tags were detected in the promoter regions of the *ACVR1C*, *ADCK5*, *EFNA2*, *ENOSF1*, *GLS2*, *KNDC1*, *MTNR1B*, *PACSIN3*, *PAX8-AS1*, *TLDC1*, and *ZNF7* genes. Using methylation evaluation, we found that *EFNA2*, *ENOSF1*, *GLS2*, *KNDC1*, *MTNR1B*, *PAX8-AS1*, and *TLDC1* showed > 5% promoter methylation in non-plaque intima, atherosclerotic vascular tissues, and buffy coats. Using logistic regression analysis, we identified hypomethylation of *MTNR1B* as an independent variable for the stenosis radiophenotype prediction model by combining it with traditional atherosclerosis risk factors including age, hypertension history, and increases in creatinine, lipoprotein (a), and homocysteine. We performed fivefold cross-validation of the prediction model using 384 patients with ischemic stroke (50 [13%] no-stenosis and 334 [87%] > 1 stenosis radiophenotype). For the cross-validation, the training dataset included 70% of the dataset. The prediction model showed an accuracy of 0.887, specificity to predict stenosis radiophenotype of 0.940, sensitivity to predict no-stenosis radiophenotype of 0.533, and area under receiver operating characteristic curve of 0.877 to predict stenosis radiophenotype from the test dataset including 30% of the dataset.

**Conclusions:**

We identified and validated *MTNR1B* hypomethylation as an epigenetic marker to predict cranial vessel atherosclerosis using stenosis radiophenotypes and blood inflammatory cells rather than direct atherosclerotic plaque sampling.

**Supplementary Information:**

The online version contains supplementary material available at 10.1186/s13148-023-01423-x.

## Background

Promoter DNA methylation changes in a specific gene are an epigenetic mechanism that causes gene silencing without genetic polymorphisms [1]. Changes in gene-specific promoter methylation may result from aging [2] and environmental influences [3]. Atherosclerosis is a disease associated with aging and environmental influences [4]. Therefore, promoter methylation profiling may be a useful epigenetic tool to understand the influence of aging and the environment on atherosclerosis development [5]. Several gene-specific promoter methylation changes related to cell proliferation and inflammation may act as epigenetic markers for atherosclerosis development [6, 7]. However, gene-specific promoter methylation events causally associated with atherosclerosis development are currently insufficient to serve as epigenetic markers for predicting the initiation and progression of atherosclerosis and cardiovascular diseases [5–7].

To identify epigenetic markers related to atherosclerosis, we addressed two criteria. First, radiophenotype characteristics are required to define the presence and severity of atherosclerosis rather than direct evaluation of atherosclerotic tissues. Molecular mechanisms in atherosclerosis development have been studied using histopathologic characteristics [8, 9]. However, in clinical practice, atherosclerosis is primarily diagnosed based on the presence and severity of stenosis as measured by angiographic and ultrasonographic tools without direct histopathological evaluations [10]. Therefore, studies using stenosis radiophenotype characteristics are critical to define the presence of atherosclerosis without direct evaluation of atherosclerotic plaques.

Second, surrogate tissues are required to replace atherosclerotic tissues. Atherosclerotic tissues are often unavailable for sampling prior to starting treatment in patients with atherosclerosis [11]. Blood inflammatory cells (monocytes, T cells, and B cells) recruit and infiltrate the vascular endothelial barrier to initiate and promote atherosclerosis in the blood vessels [12]. Thus, the blood inflammatory cells isolated from patients with atherosclerosis possess high potential as surrogate tissues for studying atherosclerosis-related epigenetic characteristics as an alternative for directly collecting atherosclerotic plaques.

The purpose of this study was to profile and validate gene-specific promoter methylation changes related to atherosclerosis using cranial vessel stenosis radiophenotypes and blood inflammatory cells instead of directly evaluating and sampling atherosclerotic plaques.

## Results

### Profile and identification of gene-specific promoter methylation changes related to stenosis radiophenotypes in blood inflammatory cells

**Fig. 1:**
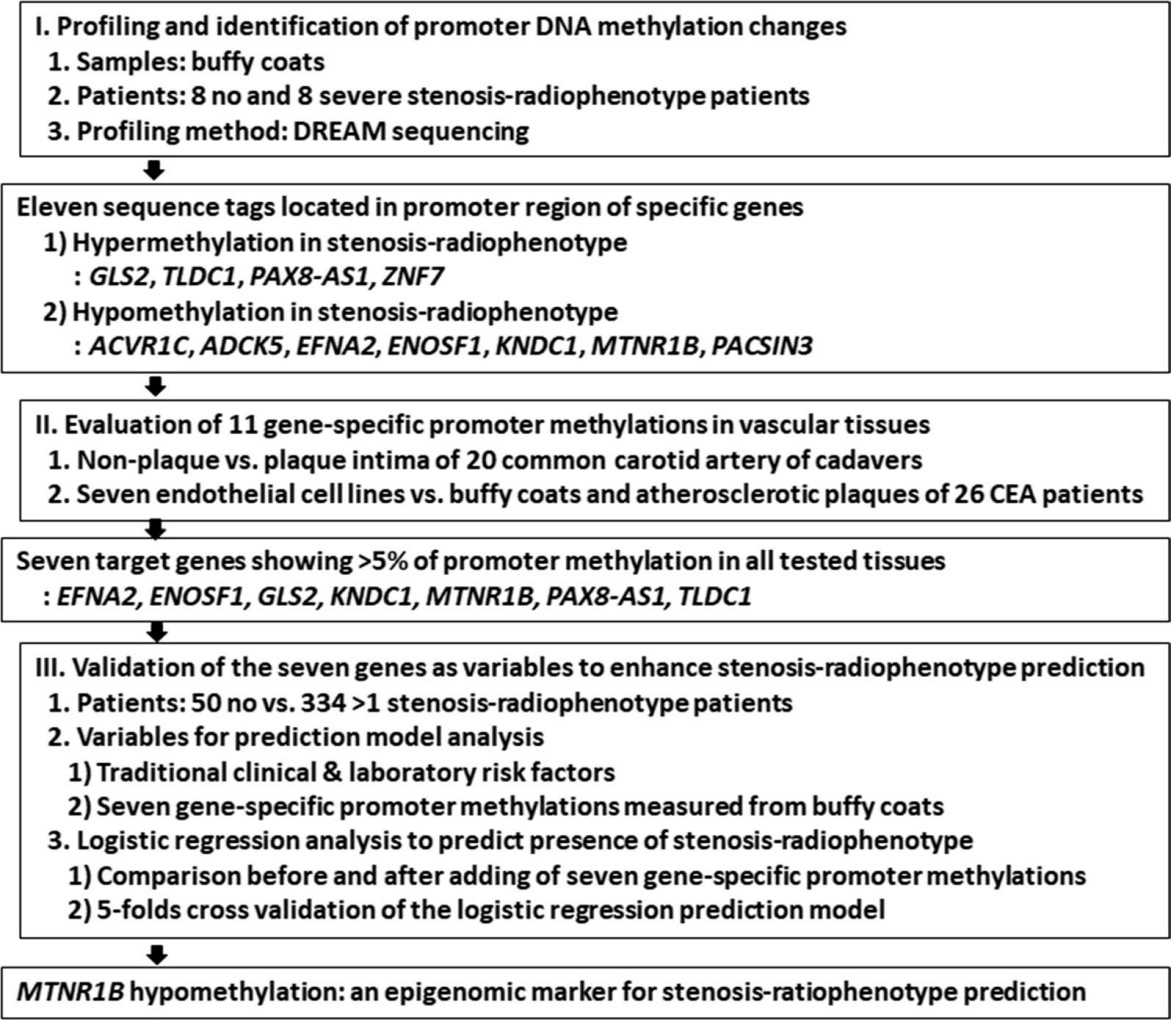
Flow diagram for profiling and validating gene-specific promoter methylation changes in cranial vessel stenosis radiophenotypes. *CCA* Common carotid artery, *CEA* Carotid endarterectomy, *DREAM* Digital restriction enzyme analysis of methylation

The present study was performed in three steps: (1) profiling and identifying gene-specific promoter methylation changes in cranial vessels using blood inflammatory cells; (2) evaluation of the identified gene-specific promoter methylation changes in vascular and atherosclerotic tissues, and blood inflammatory cells; and (3) validation of the target gene-specific promoter methylation changes (Fig. 1).

**Fig. 2:**
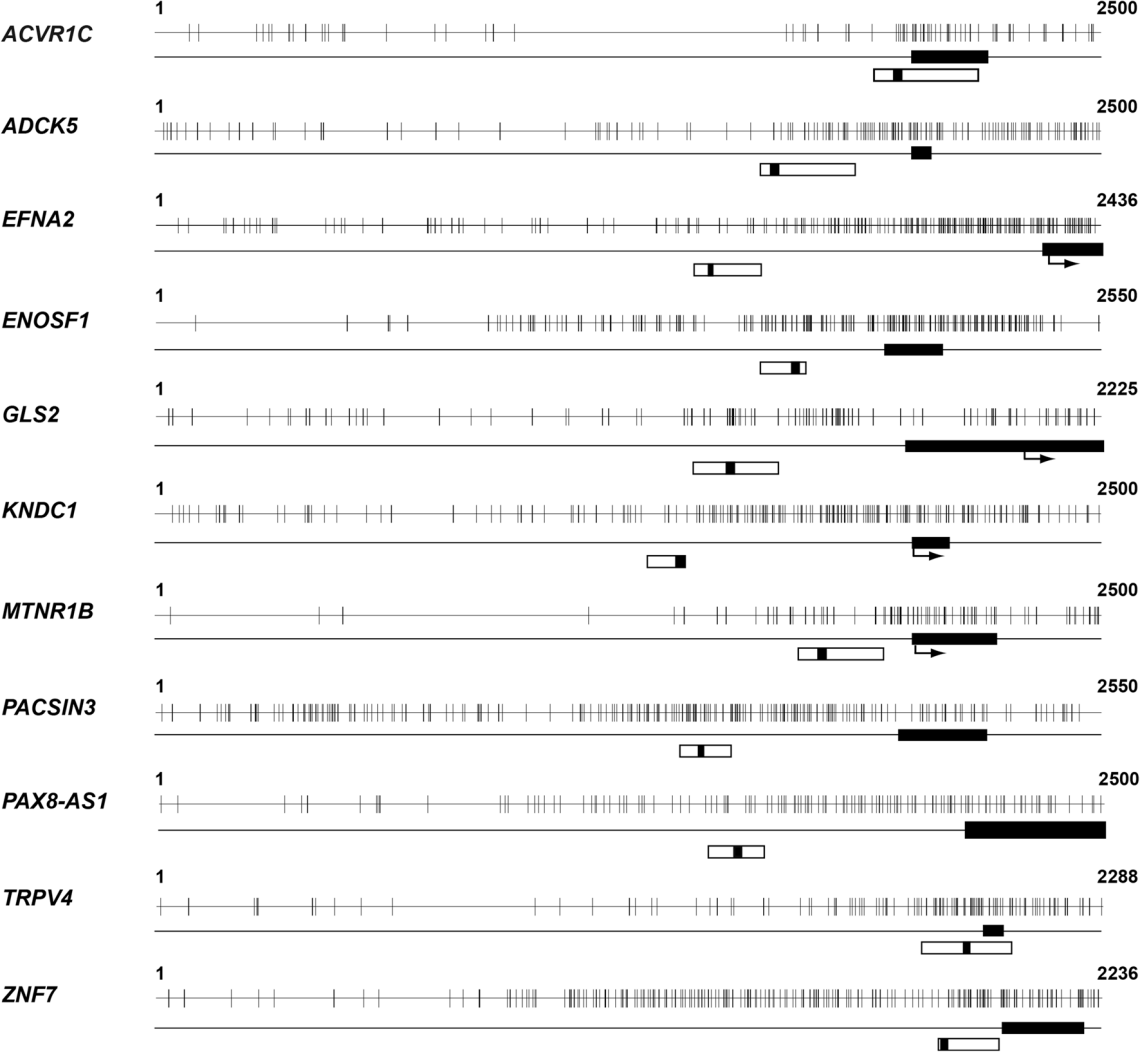
Promoter CpG islands and regions for pyrosequencing of 11 target genes identified via DREAM sequencing. *Closed bar* exon 1 region, *open bar* the regions targeted for bisulfite pyrosequencing, *closed bar in the middle of the open bar* sequencing primer region, *arrow* transcription start site of each gene

Using digital restriction enzyme analysis of methylation (DREAM) sequencing in buffy coats from eight patients each with or without severe-stenosis radiophenotypes (Additional file 1: Table S1), we identified 27,593 sequence tags with methylation differences between the two groups. We selected 138 sequence tags showing a methylation difference > 5% and *p* value < 0.1 between the two groups. Finally, we identified 11 sequence tags located ± 1000 bp from the transcription start site of specific genes (Fig. 2). Sequence tags located in the *GLS2, PAX8-AS1, TLDC1,* and *ZNF7* genes showed hypermethylation, whereas sequence tags located in the *ACVR1C*, *ADCK5*, *EFNA2*, *ENOSF1*, *KNDC1*, *MTNR1B*, and *PACSIN3* genes showed hypomethylation in patients with a stenosis radiophenotype compared with that in patients without stenosis (Additional file 1: Table S2).

### Evaluation of promoter methylation in the 11 identified genes in buffy coats, endothelial cell lines, and vascular tissues

**Fig. 3:**
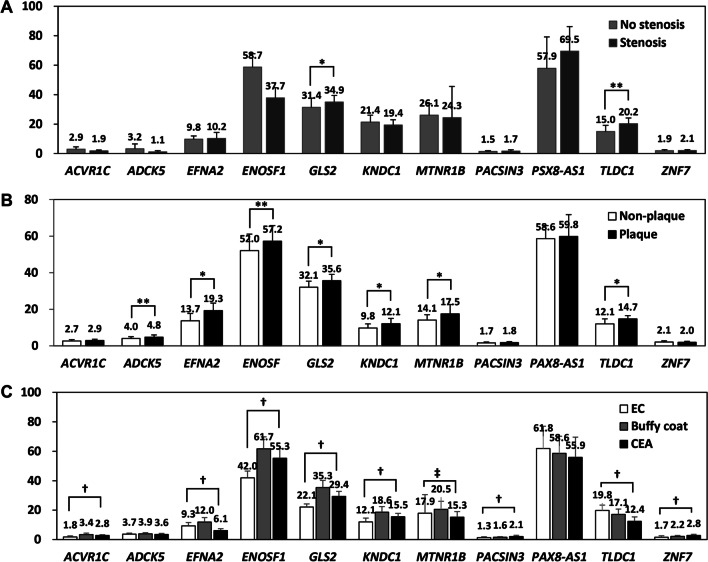
Comparison of promoter methylation levels in the 11 target genes. **A** Comparison of methylation levels between buffy coats of no-stenosis and stenosis patients included in DREAM sequencing. **B** Comparison of methylation levels between non-plaque and plaque intima of the  common carotid artery (CCA) from 20 cadavers. **C** Comparison of methylation levels between endothelial cell lines (EC), buffy coats and atherosclerotic plaques of 26 patients with carotid endarterectomy (CEA). *p < 0.01 and **p < 0.05; paired *t* test. †*p* < 0.01 and ‡*p* < 0.05; analysis of variance

By evaluating the promoter methylation of the 11 genes, we found that seven genes (*EFNA2, ENOSF1, GLS2, KNDC1, MTNR1B, PAX8-AS1*, and *TLDC1*) showed > 5% methylation, whereas four genes (*ACVR1C, ADCK5, PACSIN3*, and *ZNF7*) showed < 5% methylation in all tested vascular cell lines and tissues, and buffy coats (Fig. 3). Next, we compared non-plaque and plaque intima harvested from the common carotid artery (CCA) of 20 cadavers (Fig. 3B), and determined that *EFNA2, ENOSF1, GLS2, KNDC1, MTNR1B*, and *TLDC1* of the seven genes with promoter methylation > 5%, presented higher methylation in plaque than in non-plaque intima. Of the four genes with < 5% methylation, *ADCK5* exhibited higher methylation in plaque intima than in non-plaque intima. Comparing seven endothelial cell lines, buffy coats, and atherosclerotic plaques of 26 patients who underwent carotid endarterectomy (CEA) (Fig. 3C), seven genes with promoter methylation > 5% in CEA plaques exhibited intermediate levels of methylation in the endothelial cell lines and buffy coats. Of the seven genes with > 5% methylation, *EFNA2, MTNR1B*, and *TLDC1* showed lower methylation, whereas *ENOSF1, GLS2*, and *KNDC1* showed higher methylation in CEA plaques than in the endothelial cell lines. Of the four genes with < 5% methylation, *ACVR1C, PACSIN3*, and *ZNF7* showed statistically different methylation levels (*p* < 0.01) between the tested tissues; however, the difference was less than approximately 1%. To select target gene-specific promoter methylation events for validation analysis, we defined a methylation level > 5% as an internal control [13] and threshold for positivity [14], as previously described in pyrosequencing studies. Finally, we selected *EFNA2, ENOSF1, GLS2, KNDC1, MTNR1B, PAX8-AS1*, and *TLDC1* as target genes, which showed promoter methylation > 5% in all buffy coats, endothelial cells and vascular tissues.

### Comparison of cardiovascular risk factors and target gene-specific promoter methylation between patients with and without stenosis radiophenotype

The validation analysis was performed using 384 patients from the second set, including 50 patients with no-stenosis radiophenotype (13%) and 334 patients with > 1 stenosis radiophenotype in the cranial vessels (87%) (Table 1). Based on a univariate comparison, patients with stenosis radiophenotype (69.4 ± 8.9 years) were older than patients without stenosis (63.8 ± 8.8 years, *p* < 0.001), and displayed a mean stenosis of 4.5 in intra/extracranial vessels. Patients with stenosis radiophenotypes showed a higher frequency of past history of hypertension (*p* < 0.001) and diabetes (*p* = 0.008), and elevated levels of homocysteine (*p* = 0.049), creatinine (*p* = 0.003), lipoprotein (a) (*p* = 0.002), hemoglobin A1c (*p* = 0.04), fasting blood glucose (*p* = 0.047), and lower level of high-density lipoprotein cholesterol (*p* = 0.001) than in patients without stenosis (Table 1). Of the seven gene-specific promoter methylation events, only the *MTNR1B* gene showed significantly lower methylation levels in patients with stenosis than in those without stenosis (*p* = 0.009) (Table 1).

**Table 1:** Comparison of risk factors and laboratory tests between patients with and without stenosis

	No-stenosis (*n* = 50)	Stenosis (*n* = 334)	*p* value
Age (years, ± SD)	63.8 ± 8.8	69.4 ± 8.9	< 0.001
Sex (men/women, %)	26:24 (52%:48%)	218:116 (65%:35%)	0.069
Body mass index	25 ± 3	24.9 ± 3.2	0.819
*Stroke severity*			
NIHSS-admission	0.5 ± 1.4	0.9 ± 1.9	0.18
NIHSS-discharge	0.2 ± 0.6	0.4 ± 1	0.155
*Cranial vessel stenosis number*			
Extracranial vessels	0 ± 0	2.1 ± 1.2	
Intracranial vessels	0 ± 0	2.4 ± 2.5	
Extra- + intracranial vessels	0 ± 0	4.5 ± 2.9	
*Clinical risk factors*			
Hypertension	17 (34%)	212 (64%)	< 0.001
Diabetes	7 (14%)	108 (32%)	0.008
Smoking	12 (24%)	107 (32%)	0.252
*Blood tests*			
Hemoglobin (g/dL)	13.7 ± 1.4	13.7 ± 1.6	0.861
White blood cells (/μL)	6456.6 ± 1512.9	7027.3 ± 2105	0.066
Platelets (10^3^/μL)	234.5 ± 46.5	222.3 ± 62.2	0.184
High-sensitive C-reactive protein (mg/L)	1.6 ± 2.2	3.8 ± 9.9	0.118
Homocysteine (μmol/L)	8.7 ± 2.4	10 ± 4.7	0.049
Creatinine (mg/dL)	0.7 ± 0.2	0.8 ± 0.2	0.003
Total cholesterol (mg/dL)	181.9 ± 34.2	169.9 ± 42.2	0.056
Triglyceride (mg/dL)	130.9 ± 73.9	126.8 ± 80.4	0.738
High-density lipoprotein cholesterol (mg/dL)	51.7 ± 13.7	45.9 ± 11.3	0.001
Low-density lipoprotein cholesterol (mg/dL)	108.8 ± 27.2	102.6 ± 35.7	0.234
Apolipoprotein A (mg/dL)	134.3 ± 27.1	122.8 ± 25.1	0.003
Apolipoprotein B (mg/dL)	89.4 ± 20.3	88.4 ± 25	0.778
Lipoprotein (a) (mg/dL)	15.2 ± 11.5	25.2 ± 21.7	0.002
Hemoglobin A1c (%)	5.9 ± 0.8	6.3 ± 1.4	0.04
Fasting blood glucose (mg/dL)	113.3 ± 29.8	129.5 ± 56.2	0.047
*Gene-specific promoter methylations*			
*EFNA2*	10 ± 2.4	10.4 ± 4.1	0.456
*ENOSF1*	48.1 ± 17.7	49.6 ± 17	0.552
*GLS2*	35.6 ± 6.7	35.3 ± 6.9	0.749
*KNDC1*	21.4 ± 4.4	22.3 ± 5.2	0.276
*MTNR1B*	25.4 ± 8.7	22.7 ± 6.7	0.009
*PAX8-AS1*	63.6 ± 16.9	58.9 ± 19.7	0.105
*TLDC1*	18.3 ± 6.1	18.3 ± 5.8	0.969

*NIHSS *National Institute of Health Stroke Scale*, SD* Standard deviation

### Comparison of prediction performances before and after combining the seven gene-specific promoter methylation markers with traditional risk factors

Logistic regression analysis performed using only traditional clinical and laboratory risk factors revealed old age, hypertension history, and increases in creatinine and lipoprotein (a) levels as independent variables for the stenosis radiophenotype prediction model (Table 2). After analyzing the seven gene-specific methylation markers, increased homocysteine in the blood and hypomethylation of *MTNR1B* were identified as additional independent variables for the logistic regression prediction model. The accuracy of the model predicting the stenosis-radiophenotype before and after including the gene-specific promoter methylation changes to existing risk factors was 0.891 and 0.893, respectively, and the specificities were 0.982 and 0.973, respectively. The sensitivity to predict the no-stenosis radiophenotype was 0.28 without including the seven promoter methylations; however, this value improved to 0.36 after the inclusion. Area under the curve (AUC) of receiver operating characteristic curves of the models also improved from 0.845 to 0.880 after including the methylation variables (Table 2).

**Table 2:** Comparison of prediction performance after adding seven gene-specific promoter methylations to traditional cardiovascular risk factors

Risk factors + blood tests			Risk factors + blood tests + gene-specific methylation			Risk factors + blood tests + gene-specific methylation	
Variables	Coefficient (95% CI)	*p* value	Variables	Coefficient (95% CI)	*p* value	Important variables	Score
Age	0.057 (0.018–0.098)	0.017	Age	0.096 (0.05–0.145)	0.001	Lipoprotein (a)	100
Hypertension: Yes	1.182 (0.527–1.868)	0.004	Hypertension: yes	0.99 (0.291–1.716)	0.022	Age	93.47
Creatinine	2.864 (0.492–5.409)	0.055	Creatinine	2.727 (0.184–5.462)	0.088	Hypertension: Yes	87.81
Lipoprotein (a)	0.044 (0.023–0.071)	0.002	Lipoprotein (a)	0.048 (0.025–0.076)	0.002	*MTNR1B*	76.07
			Homocysteine	0.102 (0.016–0.218)	0.086	*PAX8.AS1*	66.5
			*MTNR1B*	− 0.099 (− 0.148 to − 0.052)	0.001	*TLDC1*	53.59
						Homocysteine	50.8
*Performance of the prediction model*							
Accuracy	0.891		0.893			0.887	
Sensitivity	0.28		0.36			0.533	
Specificity	0.982		0.973			0.94	
AUC	0.845		0.88			0.877	

*AUC* Area under the curve, *CI* Confidence interval

### Mitigation of overfitting the logistic regression model using fivefold cross-validation analysis

Using a fivefold cross-validation analysis estimated with the training dataset including 70% of the 384 patients of the second patient set, lipoprotein (a), age, hypertension history, and *MTNR1B* methylation were identified as the top four features for the prediction model (Table 2). The performance of the prediction model showed an accuracy of 0.887 and specificity of 0.940 for the test dataset, which included 30% of the second patient set. The sensitivity to predict the no-stenosis radiophenotype of the model was enhanced from 0.36 before cross-validation to 0.533 after cross-validation. The AUC of the cross-validation model for predicting the stenosis radiophenotype in the test dataset was 0.877 (Table 2).

### Methylation differences in the target genes between individual inflammatory cell types

We evaluated whether individual monocytes, T cells, and B cells composing buffy coats from 170 (28 no-stenosis radiophenotype and 142 stenosis radiophenotype) of 384 patients in the second set showed similar promoter methylation levels in the *EFNA2, ENOSF1, GLS2, KNDC1, MTNR1B, PAX8-AS1*, and *TLDC1* genes. Buffy coats showed an intermediate level of methylation for all seven genes compared with those in monocytes, T cells, and B cells (Table 3). Six of the seven genes, except for *PAX8-AS1*, showed lower methylation in monocytes than in T and B cells (Table 3). Of the seven genes, *MTNR1B* showed significantly lower methylation in buffy coats (*p* = 0.052) and B cells (*p* = 0.004), and higher levels in T cells (*p* = 0.014) from patients with stenosis than in those without stenosis. *TLDC1* showed significantly higher methylation in monocytes (*p* = 0.025) and buffy coats (*p* = 0.049) from patients with stenosis than in those without stenosis.

**Table 3:** Methylation differences in the seven target genes between inflammatory cell types

	No-stenosis (*n* = 28)	Stenosis (*n* = 142)	*p* value
*EFNA2*			
Monocyte	8 ± 3.9	9.2 ± 3.7	0.118
T cell	11.8 ± 4.1	11.9 ± 3.3	0.884
B cell	7.8 ± 3.9	9.2 ± 4.1	0.115
Buffy coat	10.3 ± 2.6	10.2 ± 4	0.901
*ENOSF1*			
Monocyte	45.3 ± 17.6	47.3 ± 16.8	0.562
T cell	47.3 ± 17.7	51.1 ± 16.3	0.268
B cell	48.7 ± 20.5	54.6 ± 16.3	0.096
Buffy coat	47.2 ± 16.8	48.9 ± 16.2	0.618
*GLS2*			
Monocyte	30.1 ± 9.2	31.5 ± 7.7	0.412
T cell	43.1 ± 7.7	43.7 ± 5.7	0.622
B cell	40.6 ± 10.4	42.5 ± 10.1	0.375
Buffy coat	36.3 ± 7.9	36.1 ± 7.4	0.944
*KNDC1*			
Monocyte	20.2 ± 5.7	21.3 ± 6.1	0.366
T cell	25.1 ± 4.6	26 ± 4.7	0.323
B cell	22.4 ± 5.2	23.1 ± 4.9	0.485
Buffy coat	21.3 ± 5.1	22.3 ± 5.4	0.383
*MTNR1B*			
Monocyte	17.4 ± 10.8	16.2 ± 6.2	0.412
T cell	37.1 ± 9.6	40.6 ± 6.8	0.02
B cell	42.1 ± 15.3	37.7 ± 8.3	0.04
Buffy coat	27 ± 10.3	23.8 ± 7.5	0.052
*PAX8-AS1*			
Monocyte	65.3 ± 21.4	62.4 ± 21.8	0.546
T cell	60 ± 15.2	58.4 ± 16.3	0.635
B cell	63.5 ± 18.5	61.9 ± 19.2	0.701
Buffy coat	63.7 ± 17.3	60.1 ± 20.6	0.393
*TLDC1*			
Monocyte	15.6 ± 5.7	18.7 ± 6.8	0.025
T cell	20.3 ± 7.4	20.6 ± 5.4	0.77
B cell	18.8 ± 7.9	20.1 ± 7.8	0.43
Buffy coat	15.7 ± 5.6	18.2 ± 6.2	0.049

Data represent mean ± SD (%)

## Discussion

We profiled epigenomic markers related to atherosclerosis using stenosis radiophenotypes and blood inflammatory cells without directly sampling atherosclerotic plaques. Hypomethylation of the *MTNR1B* promoter was identified and validated as an independent variable to predict the stenosis radiophenotype in cranial vessels by combining it with traditional clinical and laboratory risk factors. The present study demonstrates a radioepigenomic approach to identify epigenomic markers related to the presence of atherosclerosis using the stenosis radiophenotype and peripheral blood inflammatory cells.

Here, we attempted to overcome the limitations of direct evaluation and sampling of atherosclerotic plaques from the body. First, we used stenosis radiophenotypes to estimate the presence and severity of atherosclerosis. For cancer radiogenomics studies, radiophenotypes of cancer tissues are defined using imaging characteristics within and surrounding the cancer mass via computed tomography or magnetic resonance imaging. This is then used to determine relationships between molecular genomic characteristics detected in the cancer tissues [15]. Recently, radiogenomics studies of atherosclerosis have been increasing to understand the gaps in our knowledge of the disease pathogenesis, genotypes, and phenotypes without direct atherosclerotic plaque sampling [16]. To define atherosclerosis radiophenotypes, characteristics of the atherosclerotic plaque interior have been studied using multimodal imaging techniques [17, 18]. However, greater detail is required to interrogate radiophenotypes that define the composition and activity of individual atherosclerotic plaques [17, 18]. Although stenosis radiophenotypes reveal the presence and severity of atherosclerotic tissues without compositional information via magnetic resonance angiography (MRA) and carotid duplex ultrasonography (CDU), this technique has been used for medical or interventional management of cardiovascular diseases in patients [10]. We showed the potential of stenosis radiophenotypes to profile and validate the significance of gene-specific promoter methylation changes associated with atherosclerosis.

We used buffy coats containing inflammatory cells as surrogate tissues for atherosclerotic plaques. Gene-specific promoter methylation levels related to a specific disease are typically verified using tissues affected by the disease [11]. However, sampling of specific disease tissues, particularly in atherosclerosis, may be inaccessible or require sacrificing individuals [11]. Blood inflammatory cells have emerged as a surrogate tissue to study the epigenetic regulation of disease development in different tissues [19]. We profiled gene-specific promoter methylation changes related to the stenosis radiophenotype and identified 11 potential methylation markers. We observed similar patterns of methylation levels > 5% in *EFNA2, ENOSF1, GLS2, KNDC1, MTNR1B, PAX8-AS1*, and *TLDC1*, and methylation < 5% in *ACVR1C, ADCK5, PACSIN3*, and *ZNF7* in tested vascular cell lines, tissues, and buffy coats. The similar methylation patterns in the 11 target genes support the use of blood inflammatory cells as surrogate tissues to identify epigenetic alterations related to atherosclerosis.

We selected *EFNA2, ENOSF1, GLS2, KNDC1, MTNR1B, PAX8-AS1*, and *TLDC1* with methylation > 5% in both buffy coats and vascular tissues for the validation analysis. We used 5% as the lower methylation limit to select target gene-specific promoter methylation markers that were used to define the internal controls for pyrosequencing experiments [13] and efficacy of methylation phenotypes for patients with colon cancer [14]. Although *ACVR1C, ADCK5, PACSIN3,* and *ZNF7* showed a promoter methylation < 5%, the methylation of some of the promoters of these genes was statistically different between plaque and non-plaque intima or between endothelial cells and CEA plaques. However, the effects of these lower methylation limits on gene silencing and techniques to measure exact methylation levels without internal control adjustment have not been well characterized. Therefore, further studies are necessary to determine the lower limit for methylation differences that can be used as epigenetic markers for stenosis radiophenotypes in atherosclerosis.

Using logistic regression analysis, only hypomethylation of *MTNR1B* of the seven genes was identified as an independent variable for age, history of hypertension, creatinine, lipoprotein (a), and homocysteine to enhance the performance of the prediction model. Despite the similar accuracy of 0.89 and specificity of approximately 0.97 of the prediction models for predicting stenosis radiophenotypes both before and after including the gene-specific methylation, the sensitivity to predict the no-stenosis radiophenotype was enhanced to 0.36. After mitigating the overfitting from cross-validation analysis, the sensitivity of the logistic regression model was enhanced to 0.53. The enhancement in sensitivity within the dataset with 13% incidence of the no-stenosis radiophenotype suggested that including *MTNR1B* hypomethylation with traditional risk factors may provide a novel epigenetic marker for the prediction of stenosis radiophenotypes.

*MTNR1B* hypomethylation related to the stenosis radiophenotype may be an epigenetic marker to estimate the development of cranial vessel atherosclerosis. *MTNR1B* (melatonin receptor 1B) is one of the two human melatonin receptors [20]. *MTNR1B* mutations have been associated with increased blood glucose [21, 22] and risk of type 2 diabetes [23]. *MTNR1B* hypomethylation associated with the stenosis radiophenotype in the present study showed an epigenetic relationship with atherosclerosis development. Different inflammatory cell types that infiltrate into disease-affected tissues show highly heterogeneous methylation levels based on the developmental origin [24] and tissue-specific effects of the disease [25]. Atherosclerosis-related genes display distinct methylation levels in monocytes, T cells, and B cells in the blood [26]. Different *MTNR1B* methylation levels between monocytes, T cells, and B cells further confirmed epigenetic remodeling in the atherosclerosis-related genes within individual inflammatory cell types of the blood. However, further studies are critical to verify the role and cell specificity of *MTNR1B* epigenetic alterations related to atherosclerosis development.

Although we identified *MTNR1B* as an epigenomic marker for atherosclerosis using stenosis radiophenotypes and blood inflammatory cells, certain limitations prevent the radioepigenomic evaluation of atherosclerosis. First, more target CpG sites associated with atherosclerosis need to be identified in the human genome. We used DREAM sequencing that targeted over 370,000 CpG sites in the human genome reference sequence. Currently, multiple CpG sites may be targeted using tools including reduced representation bisulfite sequencing, methylated DNA immunoprecipitation, and whole genome bisulfite sequencing [27]. Despite varying target CpG numbers and densities in individual epigenome-wide association study techniques, further studies are required to identify more target CpG sites related to atherosclerosis. Next, compositional radiophenotypes are required to define the interior of atherosclerotic plaques. Currently, the stenosis radiophenotypes in the present study are primarily used as imaging markers to detect atherosclerosis presence and treat patients with cardiovascular diseases. However, to better understand the relationship between epigenetic alterations and atherosclerosis development, more details on the compositional radiophenotypes of atherosclerosis are needed. Finally, we need to collect stenosis radiophenotype data and gene-specific promoter methylation measurements from a larger number of patients. Collection of stenosis radiophenotypes and blood inflammatory cells from patients with atherosclerosis is relatively easy rather than directly evaluating and sampling atherosclerotic plaques. Therefore, the significance of the results obtained from radioepigenomic studies may be applied and validated for numerous patients. However, we must consider using the same criteria for stenosis radiophenotypes and verification of blood inflammatory cells as surrogate tissues to replace atherosclerotic plaques in future studies.

## Conclusions

We identified gene-specific promoter methylation markers associated with atherosclerosis using stenosis radiophenotypes and DREAM sequencing in blood inflammatory cells. In conclusion, the present radioepigenomic study identified and validated *MTNR1B* hypomethylation as an epigenetic marker to predict the presence of atherosclerosis using stenosis radiophenotypes and blood inflammatory cells.

## Methods

### Patients and evaluation of traditional cardiovascular risk factors

The first patient set was selected for DREAM sequencing from patients with ischemic stroke over the age of 65 years. Buffy coats from patient samples were prospectively deposited in the Human Bio-Resource Bank of the Chungnam National University Hospital from June 2013 to February 2015 (IRB review number: CNUH-2013-01-019). From this patient set, we selected eight patients with the severe-stenosis radiophenotype who had more than three vessels with > 50% stenosis in 11 intracranial vessels (middle cerebral arteries, anterior cerebral arteries, posterior cerebral arteries, intracranial internal carotid arteries, vertebral arteries of both sides, and basilar artery) on time-of-flight MRA [28] and more than two vessels with > 50% stenosis in the bilateral CCA and proximal intracranial arteries via CDU [29]. Eight patients with the no-stenosis radiophenotype who had no-stenosis of the intracranial and extracranial vessels in the two imaging studies were also selected (Additional file 1: Table S1).

A second set of patients was selected to validate the significance of the identified gene-specific promoter methylation markers in patients with ischemic stroke whose buffy coats were prospectively deposited in the Stroke Registry of the Chungnam National University Hospital from May 2017 to May 2020 (IRB review number: CNUH-2017-04-054). We selected 334 patients with the stenosis radiophenotype with > 1 stenosis and > 50% stenosis in the 11 intracranial vessels or four extracranial vessels via imaging studies, and 50 patients with no-stenosis radiophenotype in intracranial or extracranial vessels (Table 1).

Patients with ischemic stroke due to cardioembolic causes were excluded from the study. The first and second sets of patients were evaluated based on clinical cardiovascular risk factors including age, sex, body mass index, and previous history of hypertension, diabetes, and smoking, and lastly, laboratory tests including hemoglobin A1c, fasting blood glucose, total cholesterol, triglyceride, low-density lipoprotein cholesterol, high-density lipoprotein cholesterol, lipoprotein (a), apolipoprotein A and B, high-sensitivity C-reactive protein, homocysteine, white blood cell count, hemoglobin, and platelet count measured during the fasting state within 24 h after admission.

### Isolation of buffy coats and individual inflammatory cells from blood

Buffy coats were separated from blood samples (3 mL) of 18 patients in the first set after centrifugation for 15 min at 1000 rpm in EDTA-coated tubes. For 384 patients in the second set, buffy coats were separated using density gradient centrifugation (Ficoll-Paque PLUS, cat. no. 17-1440-02, Merck KGaA, Darmstadt, Germany) from blood samples (3 mL) collected in citrate-coated tubes during the fasting state within 24 h after admission. In 170 of 384 patients in the second set, T cells (Dynabeads® CD3; cat. no. 11151D, Invitrogen, USA), B cells (CD19 Pan B; cat. no. 11143D, Invitrogen), and monocytes (CD14; cat. no. 11149D, Invitrogen) were sorted from the buffy coats. All buffy coats and individual inflammatory cells from the first and second patient sets were stored at − 80 ℃ before DNA extraction. DNA from the buffy coats and individual inflammatory cell types was extracted using a kit (DNeasy Blood & Tissue Kit, Qiagen, Germany) and stored at − 20 °C until further use.

### Vascular endothelial cell culture

For wild-type endothelial cells, we cultured five human umbilical vein endothelial cell (HUVEC) lines (cat. no. C-005-5C, lot no. 1774129, Gibco, USA; cat. no. C-015-5C, lot no. 1391153, Gibco; cat. no. PCS-100-010, lot no. 70006858, American Type Culture Collection [ATCC]; cat. no. PCS-100-010, lot no. 70008844, ATCC; cat. no. PCS-100-013, lot no. 80616172, ATCC) and two human aortic endothelial cell (HAEC) lines (cat. no. PCS-100-011, lot. no. 64389694 and 70008309, ATCC). HUVECs and HAECs were cultured in M199 growth media (cat. no. 31100035, Gibco) containing less than 20% fetal bovine serum (cat. no. 12483-020, Gibco), 2% human serum, 2 mmol/L L-glutamine (cat. no. 25030-081, Gibco), and 50 μg/mL endothelial cell growth supplement (cat. no. 356006, BD Biosciences, USA). All HUVEC and HAEC lines were subcultured for up to five passages, and DNA was then extracted using a kit (DNeasy Blood & Tissue Kit, Qiagen) and stored at − 20 °C until further use.

### Collection of atherosclerotic plaque and non-plaque intima from the CCA of cadavers

We collected atherosclerotic plaque and non-plaque intima dissected from the CCA of 20 cadavers (male-to-female ratio = 14:6, age = 63.1 ± 18.8 years, mean ± standard deviation). Elevated atherosclerotic plaques in the intimal layer were distinguished from the surrounding non-plaque intima via naked-eye observation. The plaque and non-plaque regions were clearly dissected along the plaque margins, and then harvested by peeling them from the media layer of the individual CCAs and stored at − 80 °C until DNA extraction. The plaque and non-plaque intima were examined using histological hematoxylin and eosin staining. DNA from 100 mg of the formalin-fixed plaque and non-plaque intima was extracted as previously described [30], suspended in 100 μL of 1X Tris–EDTA buffer (pH 8.0), and then stored at − 20 °C until further use. The vascular tissues from cadavers were provided by the Human Resources Center in the Department of Anatomy, College of Medicine at the Chungnam National University.

### Collection of atherosclerotic tissues and buffy coats from patients who underwent CEA

Atherosclerotic plaques were harvested from 26 patients who received a CEA at Dong-A Medical Center from December 1, 2015 to September 30, 2017. All CEA plaque samples were immersed overnight in five volumes of RNAlater (Thermo Fisher Scientific, USA) immediately after collection from each patient and stored in liquid nitrogen until DNA extraction after removal of the solution. Buffy coats from each patient were simultaneously separated from blood samples (3 mL) in an EDTA tube, which was collected immediately before the CEA operation and separated after centrifugation for 15 min at 1000 rpm. DNA from 50 mg of CEA plaques and 100 μL of buffy coats were extracted using a kit (DNeasy Blood & Tissue Kit, cat. no. 69506, Qiagen) according to the manufacturer’s instructions. All CEA plaque and buffy coat samples were stored in the Human Bio-Resource Bank at the Dong-A Medical Center with informed consent.

### Profiling and mapping of gene-specific promoter methylation using DREAM sequencing

We profiled gene-specific promoter methylation related to stenosis radiophenotypes using DREAM sequencing [31] of DNA extracted from the buffy coats of eight patients in the first set each with or without the stenosis radiophenotype (Additional file 1: Table S1). In brief, a sequencing library was generated after digestion of the DNA with the SmaI and then XmaI restriction enzymes. The libraries were sequenced via paired-end 36 nt sequencing on the Illumina Genome Analyzer II or Illumina HiSeq 2000. We mapped the reads of 374,165 SmaI sites to the human genome (NCBI36/hg18) reference and signatures corresponding to methylated and unmethylated CpG genome using the Bowtie [32] and Burrows–Wheeler transform [33] aligners. Next, we excluded repetitive sequences (including LINE and Alu), and applied the following criteria to select tags that distinguish between methylation differences in no- and severe-stenosis groups: methylation difference > 5% and *p* value < 0.1. Finally, we selected target tags located in CpG islands (GC% and observed/expected ratio > 0.6) within the ± 1000 bp sequence from the transcription start site of the specific genes.

### Promoter methylation evaluation

The promoter methylation status of the 11 genes identified after the mapping analysis was evaluated using bisulfite pyrosequencing (Fig. 2). Bisulfite treatment of DNA was performed using a kit (cat. no. D5002, Zymo Research, USA) with 1 μg of DNA and stored at − 20 °C until further use. For bisulfite pyrosequencing, we used primer sets (Additional file 1: Table S3) comprising forward and reverse primer pairs for polymerase chain reaction (PCR) and a sequencing primer that amplified promoter CpG islands in individual target genes (Fig. 2). PCR for pyrosequencing of each gene was performed in a total volume of 20 μL with a premix PCR kit (AccuPower® PyroHotStart Taq PCR PreMix, cat. no. K-2611, Bioneer, South Korea) after adding 1 μL (15 ng) of bisulfite-treated DNA and 0.1 mmol/L of forward and reverse primers for individual genes, and one primer for each gene was biotinylated at the 5′-end. After denaturation for 5 min at 95 °C, the reaction cycles were as follows: 45 cycles (95 °C for 30 s, annealing temperature of individual genes for 30 s, and 72 °C for 30 s), and final annealing and extension at 72 °C for 10 min. Next, bisulfite pyrosequencing was performed using a sequencing primer for individual genes and PyroMark Gold Q96 Reagents (cat. no. 972804, Qiagen) and a pyrosequencing machine (PyroMark Q96 ID, Qiagen). Methylation levels in each gene are represented as mean values from all pyrosequenced CpG sites for individual genes.

### Statistical analysis

Differences in sex and clinical risk factors were compared using a chi-square test between no-stenosis and stenosis radiophenotypes. Differences in age, laboratory tests, and target gene-specific promoter methylation were compared between the two radiophenotype groups, vascular tissues, and inflammatory cell types using independent Student’s *t* tests. We compared the prediction performance of the logistic regression model for the stenosis radiophenotype using traditional clinical and laboratory cardiovascular risk factors. The prediction performance was modeled based on the addition of gene-specific promoter methylation markers of seven genes (*EFNA2, ENOSF1, GLS2, KNDC1, MTNR1B, PAX8-AS1*, and *TLDC1*) to the traditional risk factors in the second patient set. To mitigate overfitting of the logistic regression prediction model, we performed fivefold cross-validation analysis for the logistic regression model using the traditional clinical and laboratory risk variables, and the seven gene promoter methylation markers. For the cross-validation analysis, we first divided the second set of patients into two datasets: 70% training and 30% test datasets. Using the 70% training dataset, fivefold cross-validation of the logistic regression model was estimated. Performance of the prediction model from the 70% training dataset was validated using the 30% test dataset. The prediction performance differences before and after including the target gene-specific promoter methylation markers were measured and compared according to accuracy, sensitivity to predict the no-stenosis radiophenotype, specificity to predict the stenosis radiophenotype, and AUC. All statistical analyses were performed using SPSS (ver. 24.0, IBM Corp., USA) and R packages (ver. 4.1.3).

## Supplementary Information

**Additional file 1. Table S1.** Clinical and laboratory risk factors in patients included in DREAM sequencing. **Table S2.** Eleven target genes located ± 1000 bp from the transcription start sites of specific genes. **Table S3.** Primer sequences for pyrosequencing of the 11 candidate target genes.

## Data Availability

The datasets used and/or analyzed in the current study are available from the corresponding author upon reasonable request.

## Abbreviations

AUC: Area under the curve
CCA: Common carotid artery
CDU: Carotid duplex ultrasonography
CEA: Carotid endarterectomy
DREAM: Digital restriction enzyme analysis of methylation
HAEC: Human aortic endothelial cell
HUVEC: Human umbilical vein endothelial cell
MRA: Magnetic resonance angiography
MTNR1B: Melatonin receptor 1B
PCR: Polymerase chain reaction
