# Supplementary material for: Hypomethylation in MTNR1B: a novel epigenetic marker for atherosclerosis profiling using stenosis radiophenotype and blood inflammatory cells
Source: Clin Epigenetics. 2023 Jan 19;15:11. doi: 10.1186/s13148-023-01423-x (PMC9854223; doi:10.1186/s13148-023-01423-x)
Supplement: Supplementary file 1 — Additional file 1. Table S1. Clinical and laboratory risk factors in patients included in DREAM sequencing. Table S2. Eleven target genes located ± 1000 bp from the transcription start sites of specific genes. Table S3. Primer sequences for pyrosequencing of the 11 candidate target genes. [file 13148_2023_1423_MOESM1_ESM.docx]

**Table S1.** Clinical and laboratory risk factors in patients included in DREAM sequencing

|  | No stenosis (n = 8) | Severe stenosis (n = 8) | *p*-value |
| --- | --- | --- | --- |
| Age (years, ± SD) | 69.5±6.0 | 71.1±6.0 | 0.596 |
| Sex (Men:women) | 5:3 | 4:4 | 1.000 |
| Clinical risk factors | | | |
| Hypertension | 5 | 6 | 1.000 |
| Diabetes | 2 | 5 | 0.315 |
| Smoking | 2 | 2 | 1.000 |
| Blood tests | | | |
| Hemoglobin | 14±1 | 11.7±2.5 | 0.041 |
| White blood cells | 7221.3±2375.6 | 6521.3±2783.6 | 0.597 |
| Platelets | 214.6±67.9 | 222.4±58.1 | 0.810 |
| High-sensitive C-reactive protein | 15.1±39.6 | 9.1±18.4 | 0.706 |
| Homocysteine | 12.1±7.6 | 13.1±6 | 0.779 |
| Creatinine | 0.8±0.2 | 1.3±1.3 | 0.291 |
| Total cholesterol | 167.3±40.5 | 150.8±42.9 | 0.442 |
| Triglyceride | 148.8±72 | 109.8±77.7 | 0.315 |
| High-density lipoprotein cholesterol | 44.6±10.8 | 45.1±16.7 | 0.945 |
| Low-density lipoprotein cholesterol | 104.8±38.5 | 86.4±31.7 | 0.315 |
| Apolipoprotein A | 113.8±29.5 | 110.5±31.4 | 0.834 |
| Apolipoprotein B | 86.9±30.9 | 77.9±22.2 | 0.515 |
| Lipoprotein (a) | 12.6±9.3 | 14±7.8 | 0.754 |
| Hemoglobin A1c | 5.9±0.9 | 6.7±1.7 | 0.248 |
| Fasting blood glucose | 108±19.7 | 123.6±28.3 | 0.224 |

**Table S2.** Eleven target genes located ±1000 bp from the transcription start sites of specific genes.

| Sequence tag ID  (Sma_hg_19_) | Chromosome | | Distance  to TSS | Gene Name | Methylation | | | *p*-value |
| --- | --- | --- | --- | --- | --- | --- | --- | --- |
|  | Number | Position |  |  | Patients with stenosis | Patients without stenosis | Difference |  |
| 48906 | 2 | 158454384 | -312 | *ACVR1C* | 58.98 | 69.43 | 10.45 | 0.058 |
| 164606 | 8 | 145597073 | -658 | *ADCK5* | 55.18 | 67.57 | 12.39 | 0.026 |
| 312484 | 19 | 1285213 | -955 | *EFNA2* | 39.11 | 47.35 | 8.25 | 0.075 |
| 303539 | 18 | 712962 | -300 | *ENOSF1* | 25.97 | 40.77 | 14.81 | 0.003 |
| 223546 | 12 | 56882641 | -460 | *GLS2* | 91.3 | 81.48 | -9.82 | 0.040 |
| 281092 | 16 | 84538986 | -698 | *KIAA1609 (TLCD1)* | 64.69 | 54.23 | -10.46 | 0.051 |
| 199028 | 10 | 134973259 | -712 | *KNDC1* | 22.99 | 30.86 | 7.87 | 0.063 |
| 44961 | 2 | 113993436 | -410 | *LOC654433 (PAX8-AS1)* | 82.59 | 52.92 | -29.67 | 0.004 |
| 212087 | 11 | 92703186 | 397 | *MTNR1B* | 3.84 | 11.45 | 7.61 | 0.027 |
| 205399 | 11 | 47206960 | 472 | *PACSIN3* | 54.18 | 64.43 | 10.24 | 0.033 |
| 164846 | 8 | 146053611 | 708 | *ZNF7* | 24.09 | 17.03 | -7.06 | 0.025 |

TSS, transcription start site.

**Table S3.** Primer sequences for pyrosequencing of the 11 candidate target genes.

| Gene | Primer name | Sequence | Primer length (bp) | PCR product (bp) | Annealing temperature (℃) |
| --- | --- | --- | --- | --- | --- |
| *ACVR1C* | PCR-Forward | GGAGTGGAGGTTATTTTGTAAAA | 23 | 280 | 65.3 |
|  | PCR-Reverse | AAACCCTCCCCCAAAACA-(B) | 18 |  | 69.1 |
|  | Sequencing | TTTTTGTTGTTATTAGATGG | 20 |  | 49.9 |
| *ADCK5* | PCR-Forward | AGGTGGTGAGTTGGAGTTTTG | 21 | 257 | 68 |
|  | PCR-Reverse | ACCCCCAAAAACTTACCTTTTA-(B) | 22 |  | 66.8 |
|  | Sequencing | AGTTTTGAGGTTAAAGAGAT | 20 |  | 49.2 |
| *EFNA2* | PCR-Forward | (B)-GGGGTAGGGGTAGAGTTTTAGAGG | 24 | 183 | 61.7 |
|  | PCR-Reverse | CACTCCACATCCATCCACCTACTATACT | 28 |  | 60.8 |
|  | Sequencing | CAAAAAAACCCCAAAAAAAT | 20 |  | 44.7 |
| *ENOSF1* | PCR-Forward | (B)-TTTGGGTAGGTAGGAGGTTT | 20 | 124 | 58.2 |
|  | PCR-Reverse | CTCAAACCCCACTTCTCCCTCTAA | 24 |  | 57.9 |
|  | Sequencing | TCCATCCCAACAAAACAAAAT | 21 |  | 43 |
| *GLS2* | PCR-Forward | (B)-AGAGAGGAGGTTAAGTAAGAGT | 22 | 200 | 59.8 |
|  | PCR-Reverse | CCCAATACCTCCCTAAAATACTAATC | 26 |  | 58.7 |
|  | Sequencing | CTACTCTCCACTCAAC | 16 |  | 45.1 |
| *KNDC1* | PCR-Forward | (B)-TTGGGGTAGGAAGAGGTTAAGAG | 23 | 107 | 68.8 |
|  | PCR-Reverse | CCTCATCCCCACCTAAAAACAT | 22 |  | 70.1 |
|  | Sequencing | CCCCACCTAAAAACAT | 16 |  | 50.4 |
| *MTNR1B* | PCR-Forward | AGTTTGAGGTGGGTTAGGTATT | 22 | 238 | 61 |
|  | PCR-Reverse | CTCTCTTCCCTACCCAACT-(B) | 19 |  | 59.4 |
|  | Sequencing | GTGAGGTTTTAGTTGTTTTTGA | 22 |  | 44 |
| *PACSIN3* | PCR-Forward | GGTTTTTAGGGGGAGGGT | 18 | 137 | 56.8 |
|  | PCR-Reverse | CCCCTCCAAAACACTCAC-(B) | 18 |  | 60.3 |
|  | Sequencing | TTTAGGAGAGGTAGGATT | 18 |  | 40.3 |
| *PAX8-AS1* | PCR-Forward | TAAGTTAAGTAGTGGGATGTGGAGG | 25 | 140 | 58.5 |
|  | PCR-Reverse | AACCCAACAAAAAAATCTAACTTCTCTCT-(B) | 29 |  | 57.4 |
|  | Sequencing | AGTTTGGGTTGGGGAGAGT | 19 |  | 58.4 |
| *TLDC1* | PCR-Forward | ATTAGGTTTAGGTGGGTTTGAGAAG | 25 | 177 | 59.6 |
|  | PCR-Reverse | CTTCCCTAACACCAAATCCT-(B) | 20 |  | 60.1 |
|  | Sequencing | GGGGTGATAGGTTTAGA | 17 |  | 44.9 |
| *ZNF7* | PCR-Forward | TTGGGAGGAGGGGTTGAGA | 19 | 156 | 58.9 |
|  | PCR-Reverse | CTCACCCTCCCTAAAAACCA-(B) | 20 |  | 57.5 |
|  | Sequencing | AGGAGGGGTTGAGAATTATAG | 21 |  | 47.8 |

Bp, base pair; PCR, polymerase chain reaction
